# Coexistence of open-angle glaucoma and sarcoidosis-associated optic neuropathy

**DOI:** 10.1186/s12886-023-03104-y

**Published:** 2023-09-13

**Authors:** Eun Hye Jung, Woonghee Kim, Ra Gyoung Yoon, Ko Eun Kim

**Affiliations:** 1 Department of Ophthalmology, Nowon Eulji Medical Center, Eulji University, Seoul, Korea; 2 Department of Radiology, Nowon Eulji Medical Center, Eulji University, Seoul, Korea; 3 Department of Ophthalmology, Asan Medical Center, University of Ulsan College of Medicine, 88, Olympic-ro 43-gil, Songpa-gu, 05505 Seoul, Korea

**Keywords:** Sarcoidosis, Optic neuropathy, Neurosarcoidosis, Glaucoma

## Abstract

**Background:**

In cases with advanced glaucomatous disc changes, further changes associated with other optic neuropathies cannot be easily identified. We present a case of preexisting open-angle glaucoma and concurrent involvement of sarcoidosis-associated optic neuropathy.

**Case presentation:**

A 53-year-old man presented with gradual visual loss in his left eye, which began 1 year ago and accelerated 3 months ago. The best-corrected visual acuity in the right eye was 20/20 and counting fingers in the left. Intraocular pressures (IOP) were 12 mmHg in the right eye and 34 mmHg in the left. We diagnosed him with advanced open-angle glaucoma in the left eye based on the advanced glaucomatous cupping of the left optic disc. The IOP in the left eye dropped to 10 mmHg and was well controlled with antiglaucomatous medication; however, his left optic disc developed pallor 3 months after the treatment. The patient was revealed to be diagnosed with sarcoidosis a month ago and had been treated with systemic corticosteroids thereafter by a pulmonologist. Orbital magnetic resonance imaging revealed sarcoidosis-associated optic neuropathy in the left eye. Subsequently, optic neuropathy occurred in his right eye.

**Conclusions:**

In eyes with advanced glaucomatous disc change, detecting the coexistence of other optic neuropathies can be difficult. This report highlights the importance of careful ophthalmic examinations and investigation for etiologies of other optic neuropathies if non-glaucomatous changes are suspected even in eyes with advanced glaucoma.

## Background

Sarcoidosis is a systemic granulomatous inflammatory disease characterized by the formation of noncaseating granulomas in the affected organs [1, 2]. Although sarcoidosis predominantly affects the pulmonary system, it can affect any organ [3, 4]. Ophthalmic complications occur in 13–79% of sarcoidosis; the most common ocular manifestation is uveitis [2, 4], and optic neuropathy is the most common neuro-ophthalmic manifestations [2, 5, 6]. Involvement of optic nerve in sarcoidosis can be explained by intrinsic granulomatous infiltration, extrinsic compression, compression or infiltration of the chiasm, or raised intracranial pressure [7].

Ocular or neurologic involvement of sarcoidosis should be suspected in patients with systemic sarcoidosis who develop visual loss [2, 8]. However, identifying sarcoidosis-related optic nerve involvement in eyes with preexisting advanced glaucomatous disc changes would be difficult. Herein, we report a case of visual loss caused by preexisting advanced glaucoma and the concurrent involvement of sarcoidosis-associated optic neuropathy.

## Case presentation

**Fig. 1:**
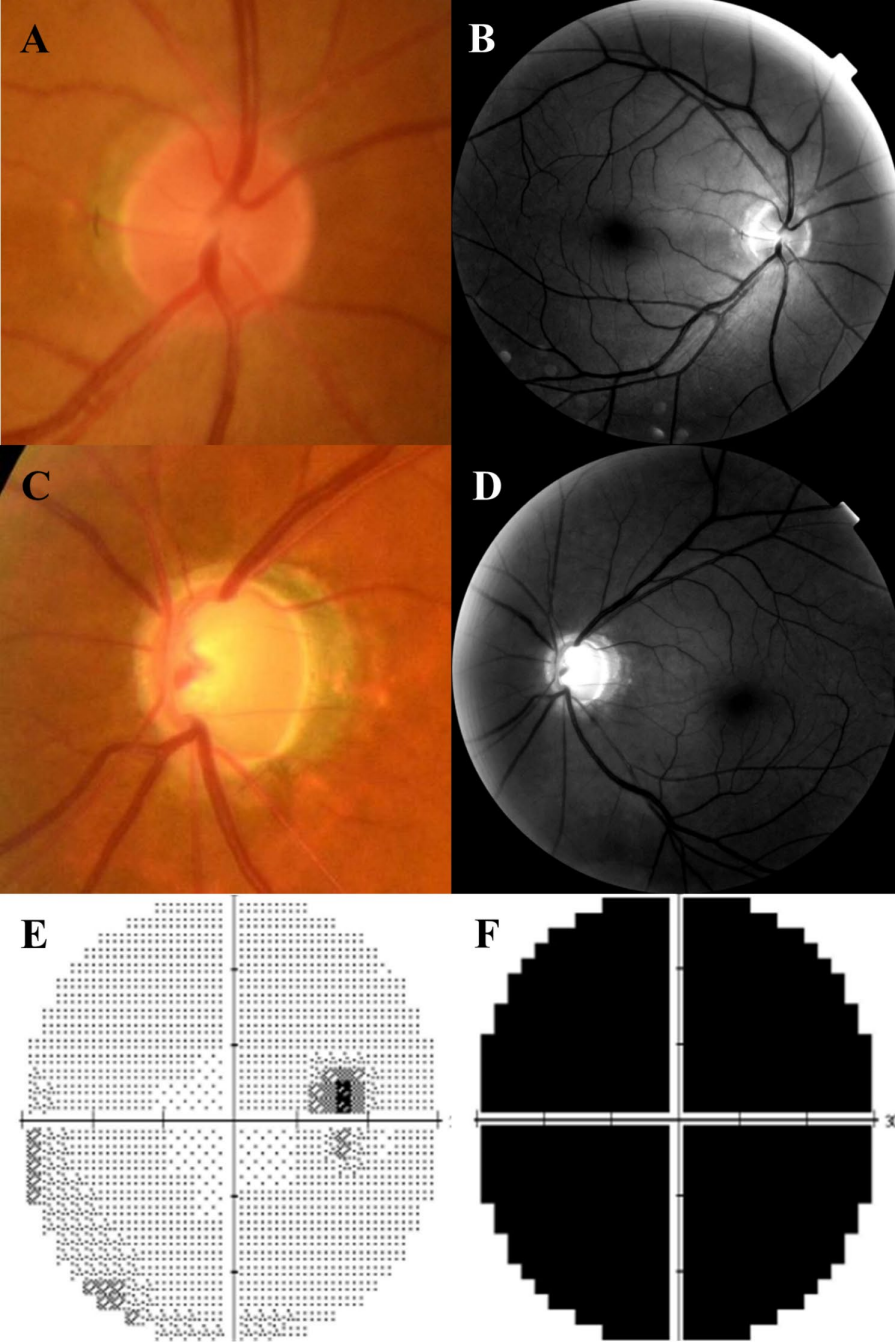
Initial examination of the patient. Optic disc photograph showing a small and crowded optic disc (**A**), and a red-free fundus photograph showing no retinal nerve fiber layer defect in the right eye (**B**). Images of the left eye showing total cupping (**C**) and diffuse superior and inferior retinal nerve fiber layer atrophy (**D**). Humphrey 30−2 visual field shows normal visual field in the right eye (**E**) but complete field loss in the left eye (**F**)

**Fig. 2:**
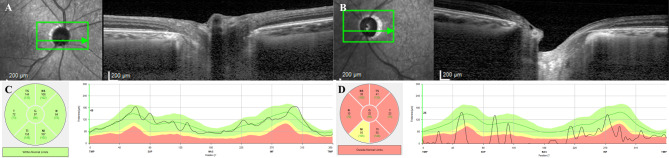
Spectral-domain optical coherence tomography images at initial visit. Horizontal optic disc scan demonstrates normal lamina cribrosa morphology and curvature in the right eye (**A**), and an increased depth of lamina cribrosa in the left eye when compared to the right eye (**B**). RNFL thickness measurement shows normal RNFL thickness profiles in the right eye (**C**), and severe RNFL loss in the left eye (**D**)

A 53-year-old man presented with gradually deteriorating vision in his left eye that began one year prior and accelerated over the last three months. At the time vision deterioration three months ago, the patient complained of periorbital pressure on his left upper eyelid. His best-corrected visual acuity (BCVA) in the right eye was 20/20, and counting fingers in the left. Intraocular pressures (IOP) measured by Goldmann applanation tonometry in the right and left eyes were 12 and 34 mmHg, respectively. Anterior segment examinations showed no abnormalities other than mild nuclear cataract in both eyes, and the gonioscopic examination using four-mirror gonioscopy lens showed an open angle with clearly visible angular structures. The right optic disc had an intact neuroretinal rim (Fig. 1A and B); however, the left optic disc had advanced glaucomatous cupping with a cup-to-disc ratio of 0.9 (Fig. 1C and 1D). Humphrey 30−2 visual field (HVF) showed normal results in the right eye (Fig. 1E) but complete field loss with a mean deviation of -32.35 dB in the left eye (Fig. 1F). Spectral-domain optical coherence tomography demonstrated an increased depth of lamina cribrosa in the left eye compared to that in the right eye (Fig. 2A and B), normal retinal nerve fiber layer thickness (RNFL) in the right eye (Fig. 2C) and diffuse RNFL loss in the left eye (Fig. 2D). There was a relative afferent pupillary defect (RAPD) in the left eye, without ocular pain or pain on extraocular movement. Pupillary light reflex in the right eye was normal. The patient was diagnosed with advanced open-angle glaucoma in the left eye and was treated with a combination of 2% dorzolamide and 0.5% timolol, 0.2% brimonidine, and 0.005% latanoprost, which lowered the IOP to 10 mmHg.

**Fig. 3:**
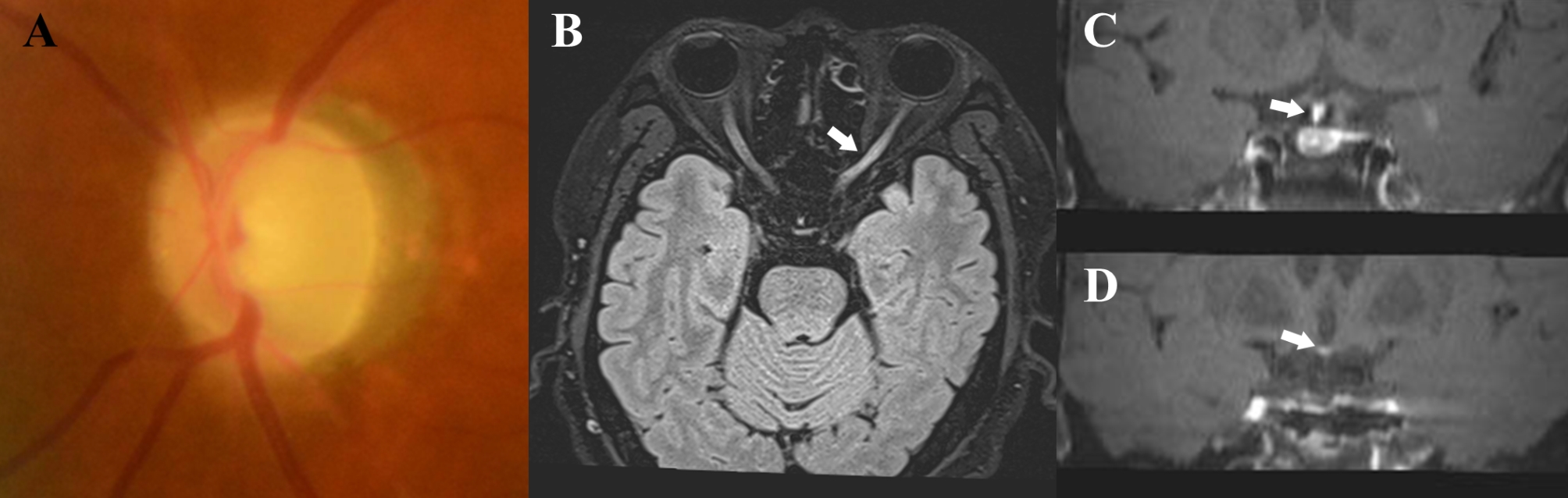
Images of the left eye at follow-up examination. At three months, a definite pallor of the left optic disc was noticed (**A**). Contrast-enhanced T2 FLAIR fat-suppressed image showing mild enhancement of the left optic nerve (**B**, arrow), and contrast-enhanced T1 SPAIR image showing mild thickening of the pituitary stalk (**C**, arrow) and right hypothalamus (**D**, arrow), as suggestive of sarcoidosis-associated optic neuropathy. FLAIR, fluid-attenuated inversion recovery; SPAIR, spectral-attenuated inversion recovery

Three months later, the IOP in the left eye was 14 mmHg. No abnormality or change was found in the anterior segment; however, pronounced optic disc pallor and thinning of the whole retinal vessels were observed in his left eye (Fig. 3A). We reexamined the patient, suspecting other concurrent optic neuropathies, and discovered that the patient had been referred to a pulmonologist due to cough and dyspnea. Approximately a month previously, he was diagnosed with sarcoidosis because he had bilateral hilar lymphadenopathy and non-caseating granulomas in the supraclavicular lymph node on radiological and histological examinations. As a result, he was treated with oral prednisolone at a dose of 24 mg/day. Although his BCVA and HVF showed no change, he expressed subjective improvement in his left peripheral visual field after treatment with corticosteroids. Additionally, we performed orbital magnetic resonance imaging (MRI) to rule out sarcoidosis-associated optic neuropathy. The images showed enhancement of the left optic nerve (Fig. 3B), a normal right optic nerve, and mild thickening of the pituitary stalk and right hypothalamus (Fig. 3C and 3D). We diagnosed that the patient had co-existing open-angle glaucoma and sarcoidosis-associated optic neuropathy in the left eye. Administration of IOP-lowering eye drops in the left eye and oral corticosteroids were maintained.

After six months of treatment, the IOP in the left eye was well controlled, and the range of IOP in both eyes was 10–14 mmHg. However, the patient complained of gradually decreasing vision in the right eye. The BCVA in the right eye was 20/40, and positive light perception was observed in the left. The IOP was 14 and 13 mmHg in the right and left eyes, respectively. Although anterior segment and funduscopic examinations and follow-up MRI images showed no changes, the HVF showed mild peripheral depression in the right eye.

**Fig. 4:**
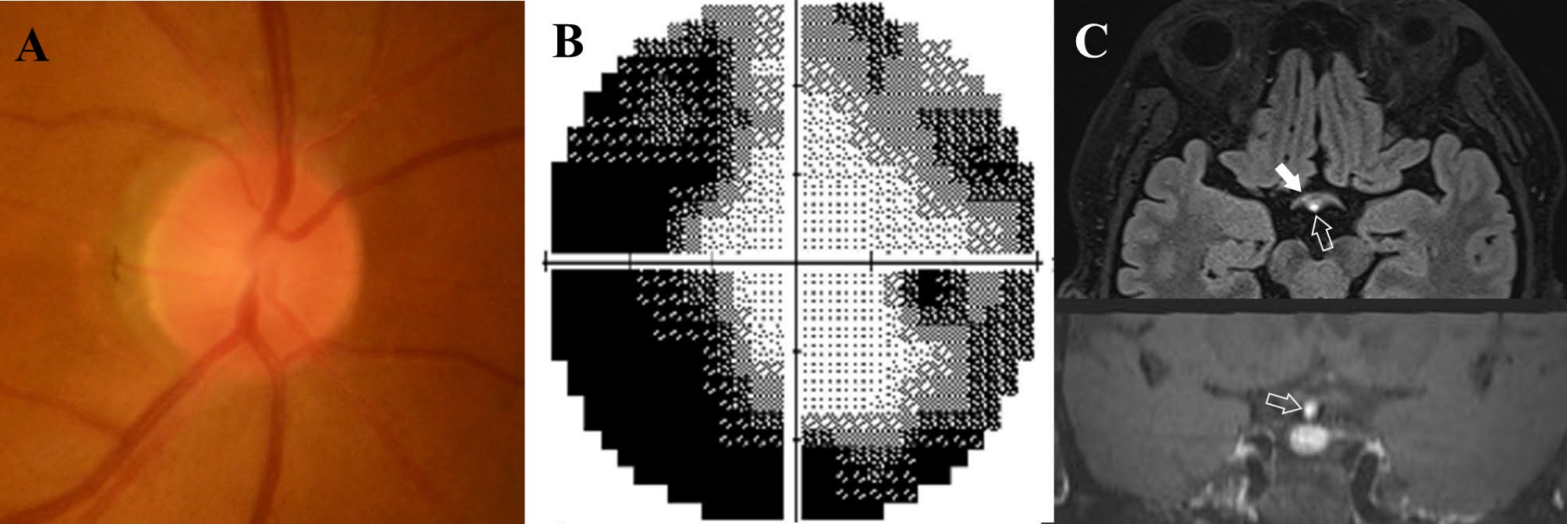
Images of the right eye at follow-up examination. After 10 months of corticosteroid treatment, despite the optic disc photograph of the right eye showing no change (**A**) compared with that at the initial exam, Humphrey 30−2 visual field shows generalized peripheral depression in the right eye (**B**). Follow-up orbital magnetic resonance imaging demonstrates newly developed focal enhancement near the junction of the right optic nerve and chiasm (**C**, upper panel, filled arrow) and enhancement of the pituitary stalk (**C**, upper panel, blank arrow; see higher magnification image with an arrow in the lower panel), suggestive of sequential sarcoidosis-associated optic neuropathy

After 10 months of corticosteroid treatment, he discontinued this medication owing to its side effect, arthralgia. After ceasing medication for two months, his BCVA decreased to 20/60 in the right eye and positive light perception was maintained in the left. Anterior segment and fundus examination showed no signs of sarcoidosis-associated uveitis (Fig. 4A), and fluorescein angiography presented no signs of active inflammation or abnormal leakage. However, the HVF showed aggravated peripheral depression in the right eye (Fig. 4B). Additionally, MRI showed newly developed focal enhancement near the junction of right optic nerve and chiasm (Fig. 4C). Laboratory tests revealed no abnormal serum autoimmune antibodies. We diagnosed the patient with sarcoidosis-associated optic neuropathy in the right eye and restarted corticosteroids at a dose of 40 mg/day and methotrexate at 7.5 mg/week. Ten months after re-treatment (in total, 23 months of follow-up), the BCVA in the right eye improved to 20/50, while in the left, positive light perception was maintained.

## Discussion and conclusions

Here, we present a case of isolated sarcoidosis-associated optic neuropathy with pre-diagnosed open-angle glaucoma. Our case showed development of prominent non-glaucomatous disc changes (generalized disc pallor) associated with sarcoidosis-associated optic neuropathy, distinctive of pre-existing glaucomatous cupping and neuroretinal rim thinning. Moreover, the sequential and progressive nature of the bilateral involvement in this case of sarcoidosis-associated optic neuropathy highlights the importance of careful ocular examinations during the course of disease.

Sarcoidosis can affect any segment of the eye and orbital structures [9]. Ocular hypertension and glaucoma can also occur in patients with sarcoidosis; however, they are usually secondary, as a result of complications from trabecular meshwork dysfunction due to edema or obstruction from inflammatory cells in sarcoidosis [9]. Our patient showed no inflammatory signs of sarcoidosis-associated changes on anterior segment nor fundus examinations at initial visit and follow-up examinations. Some could argue that the preexisting disc damage in our patient may not be solely glaucomatous. The presence of periorbital pressure sensation, subacute and progressive visual loss to finger count, and RAPD could have indicated the concurrent involvement of non-glaucomatous optic neuropathy. We could not ascertain the diagnosis due to lack of previous ophthalmologic data in our patient. However, we speculated on a high probability of preexisting glaucoma based on the following signs: (1) elevated IOP without evidence of optic nerve or orbital compression or inflammatory causes and (2) definite glaucomatous-like disc cupping with rim thinning probably caused by elevated IOP, with greater depth of lamina cribrosa and prelaminar thinning compared to the contralateral eye.

Sarcoidosis of the nervous system (neurosarcoidosis) occurs in 5–16% of patients with sarcoidosis and involves cranial nerves, anterior visual pathway, and pituitary-hypothalamic regions [2, 8, 10, 11]. Cranial neuropathy is the most frequent manifestation, and the optic nerve is the second most commonly involved among the cranial nerves [8, 12]. Although clinical manifestations of sarcoidosis-associated optic neuropathy have resembled optic neuritis in general, several studies have shown a progressive chronic change over the course of the disease [5]. Unilateral optic nerve involvement appears to be relatively more common, but bilateral synchronous or sequential involvement after a latency period of one to six months can also occur [5, 12]. Our case showed bilateral sequential involvement of optic neuropathy with a progressive course. Furthermore, as presented in the right eye of our patient, eyes with neurosarcoidosis may be undiagnosed when accompanying a normal-looking fundus [7]; therefore, suspicion and thorough examinations in both eyes are essential in patients with systemic sarcoidosis.

Neurosarcoidosis is diagnosed based on the clinical manifestations and diagnostic evaluation, which includes MRI, cerebrospinal fluid, and electromyogram or nerve conduction study findings that are typical of granulomatous inflammation of the nervous system, as well as the rigorous exclusion of other causes [8]. The diagnosis for neurosarcoidosis is based on the currently accepted diagnostic criteria that classify neurosarcoidosis cases into three categories: “definite” (positive histology of the neural tissue), “probable” (positive histology of the nonneural tissue), and “possible” (no histological support) [8]. Our patient was diagnosed with probable neurosarcoidosis based on the evidence of vision loss and abnormal MRI findings, along with the pathological confirmation of systemic sarcoidosis and exclusion of other possible causes.

The most common MRI findings of sarcoidosis-associated optic neuropathy is diffuse enlargement of the optic nerve (granulomatous infiltration) as well as thickening and enhancement of the optic nerve dura [2, 7]. Extensive optic nerve enhancement and noncontiguous nodular involvement of the contralateral nerve are also suggestive of sarcoidosis [7]. The MRI findings of the optic nerve in our case were subtle and not those commonly found in sarcoidosis-associated optic neuropathy. However, enhancement within the optic nerve and pituitary stalk and hypothalamic involvement along with other clinical manifestations can be suggestive of neurosarcoidosis.

Treatment of ocular or neuro-ophthalmic manifestations of sarcoidosis remains empiric, mainly with corticosteroids initially followed by steroid-sparing immunosuppressants [2, 13]. Methotrexate has been the most widely used steroid-sparing agent for sarcoidosis [14]. The majority of the previous studies have reported good responses to such treatments in patients with neurosarcoidosis involving the optic nerve [2, 5]. However, contradicting results were found in our case. We speculated that this may have been attributed to the delayed and insufficient initial dose (0.3 mg/kg/day) of prednisolone administered in this case. Typically, for pulmonary sarcoidosis, it is suggested to initiate treatment with oral prednisolone at a dose of 20–40 mg/day [3]. However, neurosarcoidosis necessitates a recommended dose of 0.5–1 mg/kg/day or 40 mg/day [2, 5, 12, 13]. It is important to note that if neurosarcoidosis is present, more prolonged higher-dose corticosteroid treatment is required from the outset [5]. Moreover, neurological manifestations caused by acute inflammation respond better to corticosteroids than those caused by chronic fibrotic reactions [5]. Our case, which had a more progressive disease course with sequential bilateral involvement, might have required prolonged high-dose corticosteroids and immunosuppressants from the outset. Additionally, neurosarcoidosis involving the central nervous system is reported to show less treatment response [12].

Systemic corticosteroid for sarcoidosis treatment can cause steroid-induced IOP elevation or glaucoma based on the dose, frequency, and duration of corticosteroid use [15]. Therefore, the IOP and optic disc should be monitored carefully during the course of treatment. As our patient had no history of corticosteroid treatments (ocular and systemic) and no definite IOP elevation in both eyes during corticosteroid treatment, we excluded the diagnosis of steroid-induced glaucoma.

In eyes with advanced glaucomatous damage, other optic neuropathies can be easily missed. However, non-glaucomatous changes are distinctive even in eyes with a preexisting advanced glaucomatous disc change. In such cases, careful ophthalmic examinations as well as thorough investigation for underlying systemic etiologies should be considered. Moreover, since sarcoidosis-associated optic neuropathy can affect both eyes, regular follow-ups with bilateral examinations may be necessary.

## Data Availability

All the data supporting our findings is contained within the manuscript.

## List of abbreviations

BCVA: Best-corrected visual acuity
FLAIR: Fluid-attenuated inversion recovery
HVF: Humphrey visual field
IOP: Intraocular pressure
MRI: Magnetic resonance imaging
SPAIR: Spectral-attenuated inversion recovery
